# Urologic Complication after Laparoscopic Hysterectomy in Gynecology Oncology: A Single-Center Analysis and Narrative Review of the Literature

**DOI:** 10.3390/medicina58121869

**Published:** 2022-12-18

**Authors:** Vito Andrea Capozzi, Luciano Monfardini, Elisa Scarpelli, Giuseppe Barresi, Isabella Rotondella, Alessandra De Finis, Davide Scebba, Giuseppe Maglietta, Stefano Cianci, Tullio Ghi, Roberto Berretta

**Affiliations:** 1 Department of Medicine and Surgery, University Hospital of Parma, 43125 Parma, Italy; 2 Clinical and Epidemiological Research Unit, University Hospital of Parma, University of Parma, 43125 Parma, Italy; 3 Unit of Gynecology and Obstetric, Department of Human Pathology of Adult and Childhood “G. Barresi”, University of Messina, 98125 Messina, Italy

**Keywords:** minimally invasive surgery, urologic complications, hysterectomy, gynecologic oncology

## Abstract

*Background and Objectives:* Minimally invasive surgery (MIS) has recently increased its application in the treatment of gynecological malignancies. Despite technological and surgical advances, urologic complications (UC) are still the main concern in gynecology surgery. Current literature reports a wide range of urinary tract injuries, and consistent scientific evidence is still lacking or dated. This study aims to report a large single-center experience of urinary complications during laparoscopic hysterectomy for gynecologic oncologic disease. *Materials and Methods:* All patients who underwent laparoscopic hysterectomy for gynecologic malignancy at the Department of Medicine and Surgery of the University Hospital of Parma from 2017 to 2021 were retrospectively included. Women with endometrial cancer, cervical cancer, ovarian cancer, uterine sarcoma, or borderline ovarian tumors were included. Patients undergoing robotic surgery with incomplete anatomopathological data or patients lost during follow-up were excluded from the analysis. Intraoperative and postoperative UC were analyzed and ranked according to the Clavien-Dindo classification. *Results:* Two hundred-sixty patients were included in the study: 180 endometrial cancer, 18 cervical cancer, nine ovarian cancer, two uterine sarcomas, and 60 borderline ovarian tumors. Nine (3.5%) UCs were reported (five intraoperative and four postoperative complications). No anamnestic variables showed a statistical correlation with the surgical complication in the univariable analyses. C1 radical hysterectomy, a higher FIGO stage, and postoperative adjuvant treatment (*p*-value = 0.001, *p*-value = 0.046, and *p*-value = 0.046, respectively) were independent risk factors associated with the occurrence of UC. *Conclusions:* The urological complication rates in patients with oncological disease are relatively rare events in the expert hands of dedicated surgeons. Radical hysterectomy, FIGO stage, and adjuvant treatment are independent factors associated with urinary complications.

## 1. Introduction

In the past three decades, minimally invasive surgery (MIS) has impressively increased its use in gynecologic oncology. A short time of recovery, less post-operative pain, a decrease in analgesic drug consumption, minor blood loss, and low rates of wound infections are only a few benefits of laparoscopic procedures in all gynecological malignancies [1].

### 1.1. Endometrial Cancer

Endometrial cancer (EC) was the first gynecological cancer to which MIS was applied. In 1993, Childers et al. [2] first reported two cases of laparoscopic-assisted vaginal hysterectomy. After that, a large amount of scientific evidence confirmed laparoscopic surgery as safe and feasible in EC patients with overlapping oncologic outcomes compared to open surgery [3,4,5]. Since the 2000s, MIS has represented the gold standard in the surgical treatment of endometrial cancer [6]. Furthermore, the laparoscopic sentinel lymph node algorithm became a milestone for EC surgical staging, also in high-risk tumors [7], avoiding complications related to extended lymphadenectomies [8]. This aspect also reinforced laparoscopic use in EC cases, and laparoscopic near-infrared optics with Indocyanine green tracer is strongly recommended by international guidelines [7,9].

### 1.2. Ovarian Cancer

MIS applications in ovarian cancer (OC) are varied and have different purposes depending on the FIGO stage addressed [10]. In the advanced International Federation of Gynaecology and Obstetrics (FIGO) stage, laparoscopy allows evaluation of peritoneal surfaces with tumor burden assessment by Fagotti’s score [11]. In addition, peritoneal biopsy for histologic determination and somatic breast cancer (BRCA) mutational status is crucial for a proper treatment course. Data on laparoscopic primary debulking surgery in advanced OC are still preliminary and feasible only in experimental clinical trials [12,13]. Therefore, international guidelines consider laparotomy as the standard of care for advanced OC [14]. The MIS’s role in interval debulking surgery is currently under investigation. Retrospective data suggest surgical feasibility in tertiary centers with experienced surgeons and patients with complete responses to neoadjuvant chemotherapy [15]. However, clarification will be provided by the ongoing LANCE trial [16]. Finally, MIS in early-stage OC can be considered by trained surgeons in expert centers and in cases with small pelvic masses where cyst rupture should be avoided [17]. Furthermore, laparoscopy is provided by guidelines in cases of incidental OC findings for restaging purposes [14].

### 1.3. Borderline Ovarian Tumor

The guidelines for borderline ovarian tumor (BOT) treatment follow those for early-stage OC. BOT cases have a higher prevalence in young women and account for about 10% of ovarian tumors [18]. According to recent guidelines [14], unilateral salpingo-oophorectomy and multiple peritoneal biopsies represent the standard treatments. Furthermore, young patients with small tumor sizes and early-stage disease are suitable for MIS or ultra-minimally invasive approaches without differences in oncologic outcome compared to open surgery [19].

### 1.4. Cervical Cancer

After decades of laparoscopic treatment of cervical cancer (CC) cases, a laparoscopic approach to cervical cancer (LACC) trial completely changed the previous standard of care. Indeed, the authors reported a 4.5-year disease-free survival rate of 86.0% with minimally invasive surgery and 96.5% with open surgery (95% confidence interval, −16.4 to −4.7) [20]. After the trial was published, a dramatic decline in MIS in CC treatment was recorded [21], and currently, laparoscopic hysterectomy can be considered only in extremely selected cases with microscopic diseases (FIGO stage IA) or after conization with healthy margins and absence of residual disease [22].

### 1.5. Urologic Issue

Despite the technological and surgical advances in laparoscopic surgery over the years, urologic complications (UC) are still the main concern of every gynecologic oncologist [23]. The anatomic and embryological correlation of the reproductive and urinary systems, the distorted tissue planes associated with cancer, and adhesions are factors that could complicate every oncological procedure. Urinary tract injuries include both intraoperative (bladder or ureteral section, ligation, or coagulation) and postoperative complications (bladder or ureteral fistulas, micturition difficulties, incontinence, urinary retention, and urinary tract infection) [24]. Current literature reports a wide range of urinary tract injuries in gynecology oncology, ranging from 0.8% to 14% [23,24,25]. In a large series of 317 laparoscopy radical procedures, the incidence of bladder injury was 2.2%, while the ureter injury rate was 1.1% [26]. A prompt intraoperative diagnosis of urinary lesions allows for easier complication management, while post-operative diagnosis could be difficult and delayed. Furthermore, postoperative complications can occur during hospitalization but, in some cases, also after patients’ discharge [27]. According to two studies [26,27,28], the incidence of vesicovaginal fistulas in patients who underwent laparoscopic radical hysterectomy was about 2%, while post-operative urinary retention rates were about 14% [28]. However, similar rates are reported also after open radical hysterectomy [28].

Much has changed since Ernst Wertheim called urologic complications during radical hysterectomy an “unavoidable evil” in 1898 [29]. However, consistent scientific evidence on urologic complications during laparoscopic oncological hysterectomy is still lacking or outdated. Moreover, most of the studies are focused on laparoscopic radical hysterectomy, which nowadays is seldom performed.

This study aims to report a large single-center experience of urinary complications during laparoscopic hysterectomy for gynecologic oncologic pathology. Primary objective was to quantify the laparoscopic complications rate in gynecologic oncologic patients. As a secondary objective, potential risk factors associated with intra and postoperative complications were further investigated.

## 2. Methods

All patients who underwent laparoscopic hysterectomy for gynecologic malignancy at the Department of Medicine and Surgery of the University Hospital of Parma from January 2017 to December 2021 were retrospectively included in the analysis. Women with endometrial cancer, cervical cancer, ovarian cancer, uterine sarcoma, or borderline ovarian tumors were included. Patients’ clinical data and previous surgeries were collected as reported in Table 1. Complete surgical staging was performed based on the primary disease following the European Society of Gynaecological Oncology (ESGO) and National Comprehensive Cancer Network (NCCN) international guidelines. Surgical procedures and types of hysterectomy are shown in Table 2. Patients undergoing robotic surgery with incomplete anatomopathological data or patients lost during follow-up were excluded from the analysis. The case history of a single surgeon (R.B.) with two decades of experience in gynecologic oncologic surgery was reported.

UC included both bladder (injuries, urinary retention, urinary incontinence, fistulas) and ureteral (injuries, ureteral obstruction, fistulas) complications occurring both intraoperatively and postoperatively. A superficial bladder injury included involvement of the serosal or muscular bladder layer. Deep bladder injuries included full-thickness involvement of the bladder wall (serosa, muscular, and mucosal layers). Clavien Dindo classification was used to rank surgical complications (types of surgical complications and treatments are reported in Table 3). Diagnosis of complications was performed intraoperatively by the surgeon or by computed axillary tomography or ultrasound during the postoperative period (within 6 weeks after surgery).

All surgical procedures were performed by dedicated gynecologic oncology surgeons. The ethics committee of the University Hospital of Parma approved the following study under code: 23/2022/OSS/AOUPR.

### Statistical Analysis

Descriptive statistics were used to compare the baseline demographic and clinical characteristics of the study cohort. Continuous variables are presented as median and interquartile range (IQR), and categorical variables are expressed as the absolute (number) and relative (percentage) of patients. Percentage of complications is expressed as absolute (number) and relative (percentage) of patients with Poisson 95% confidence intervals. Univariable logistic regression analysis was used to evaluate the relationship between covariates and outcomes. All of the tests were two-sided, and a *p*-value of 0.05 was considered statistically significant. The statistical analyses were made using R Statistical software, version 4.2.1. (R Foundation for Statistical Computing, Vienna, Austria).

## 3. Results

Two hundred sixty patients with a mean follow-up of 36.3 months met the inclusion criteria. In the entire series, 180 endometrial cancer cases, 18 cervical cancer cases, nine ovarian cancer cases, two uterine sarcoma cases, and 60 borderline ovarian tumor cases were analyzed. Nine (3.5%, 95% confidence interval (CI) 1.6–6.6) urinary complications were reported. Of the five intraoperative complications, three superficial bladder injuries, one full-thickness bladder injury, and one ureteral resection were reported. The four postoperative complications consisted of one ureteral kneeling with hydronephrosis, one vesicovaginal fistula, one ureteral fistula, and one urinary retention. Intraoperative bladder lesions were repaired by a double (if superficial) or triple-layer (if full-thickness) 3-0 vicryl suture. Ureteral resection required ureter-ureteral anastomosis and double-j stent placement. For postoperative complications, a double-j stent was used to correct ureteral kneeling and a ureteral fistula. A Martzius flap was used to repair the vesicovaginal fistula. Finally, urinary retention was treated conservatively with bladder catheter placement for 10 days.

Univariable analyses are summarized in Table 4, in which any clinical and anamnestic variables were statistically associated with the occurrence of complications. No clinical and anamnestic variables showed a statistical correlation with surgical complication; however, previous urological surgery (OR 1.98, 95% CI: 0.72–3.92, *p* = 0.082) and CC as the primary disease (OR: 4.20, 95% CI: 0.59–19.1, *p* = 0.089) were close to the statistical significance. On the other hand, C1 radical hysterectomy, a higher FIGO stage, and postoperative adjuvant treatment were founded as three independent risk factors associated with the occurrence of complications (OR: 23.6, 95% CI: 2.79–167, *p*-value = 0.001, OR: 3.98, 95% CI: 0.95–15.7, *p*-value = 0.046, and OR: 4.20, 95% CI: 1.08–20.3, *p*-value = 0.046, respectively). Moreover, observed as risk factors close to statistical significance were pelvic and aortic lymphadenectomy, LVSI, and tumor grading variables (*p*-values range from 0.1 to 0.12).

## 4. Discussion

### 4.1. Study Finding

The study reported a 3.5% rate of urologic complications during laparoscopic hysterectomy for gynecologic oncologic disease. Moreover, urinary complications were associated with the radical hysterectomy, the advanced FIGO stage, and cases requiring adjuvant treatment. Finally, patients who underwent previous urologic surgery and women affected by CC showed an increased likelihood of urological surgical complications.

### 4.2. Interpretations

A low urological complications rate has been achieved over the years by increasing attention to the nerve-sparing approach during hysterectomy and dedicated surgeon training for oncological disease [30]. The opening of the paravesical and pararectal spaces, identification of the ureteral course, and recognition of nerve plexuses during every procedure allowed for an extremely low complication rate, also in the context of oncological surgeries [31]. In addition, as widely reported in the literature, surgeons dedicated to oncologic pathology ensure greater surgical radicality with lower intra- and postoperative complication rates [32].

Despite this, our study reported an increased risk of urological complications in patients undergoing radical hysterectomies compared to patients who underwent non-radical hysterectomies. The anatomical variability of the pelvis, the proximity of the ureter to the uterine artery, and the need to remove parametrial tissue are factors that make the procedure extremely complex. Consistent with our results, Likić-Ladević et al. [33] reported a UC rate of 3.7% in a large retrospective series involving 536 abdominal hysterectomies. In addition, Uccella et al. [28] showed 8% urinary tract injuries in patients undergoing laparoscopic radical hysterectomy compared to the 4.2% UC in the laparotomy group, even if statisical significance was not achieved (*p* = 0.68). Moreover, Hwang [23], in a previous meta analysis, including 20 studies in 2012, reported 6.0% urologic complications during radical hysterectomy, and this risk was almost double compared to the laparotomic approach (OR 1.97, 95% CI 1.23–3.13). Therefore, in light of these data and the recent LACC trial, laparoscopic radical hysterectomy is a known risk factor for UC as well as unfavorable oncologic survival.

The study also reported a higher UC rate in patients undergoing laparoscopic hysterectomy for an advanced FIGO stage or needing adjuvant treatments. Both of these aspects are related to each other, as an advanced FIGO stage requires adjuvant treatment. As is known, an advanced disease requires more aggressive surgical procedures such as a complete ureteral roll during a radical hysterectomy [28]. Although ureteral displacement can be performed carefully, ureteral capillaries or arterioles can be damaged or clotted, decreasing ureteral vascular supply and increasing the risk of fistulas [34,35]. Then, ureteral rolling could damage the ureteral sheath and mesoureter, leading to hypogastric nerve injury with worsening ureteral peristalsis and detrusor contraction [36]. Furthermore, larger uterine tumors or enlarged ovarian masses decrease the space between the lesions and the urinary structures, increasing the risk of ureteral or bladder injury [37,38].

Finally, in our series, extracorporeal lithotripsy was the most frequently performed procedure in patients with UC after hysterectomy. The lithotripsy shock waves traumatize the urinary tract with an inflammatory response that could change the normal retroperitoneal anatomy by creating tenacious adhesions between the urinary and gynecological systems [39]. Thus, increased attachment of the ureter and bladder to the gynecological system (such as the ovaries or infundibulopelvic ligament) would require more traction, cutting, and coagulation with an increased risk of urologic injury [40].

### 4.3. Clinical Implication and Previous Literature

An update on urologic complications in gynecologic oncologic surgery is needed for better patient counseling and further improvement of surgical techniques. Moreover, recognizing UC risk factors allows for greater caution in surgical dissection or the use of precautions such as preoperative ureteral stent placement in selected patients. In line with this hypothesis, Schimpf et al. reported ureteral stent placement could also result in cost savings in selected patients requiring radical surgery [41]. In a decision-tree analysis of clinical scenarios (use universal ureteral catheterization vs. no catheterization), the authors reported that universal ureteral catheterization was cost-saving when the rate of ureteral injuries was greater than 3.2%. In a large meta-analysis, Feng et al. also reported that prophylactic placement of a ureteral catheter reduced ureteral injury, shortening the operative time [42]. On the other hand, Liang et al. reported that preoperative stenting did not reduce ureteral stricture in cervical cancer patients undergoing radical surgery followed by radiation therapy [43]. As a viable alternative, in cases of radical surgery with the need for extensive ureteral lateralization, ureteral stent placement can be performed at the end of the surgical procedure, avoiding universal ureteral catheterization that is certainly unnecessary for most patients.

To summarize some surgical expedients that could reduce the risk of urological complications, a narrative review of the literature is given below.

### 4.4. Post-Hysterectomy Cystoscopy

Some authors suggest universal post-hysterectomy cystoscopy for early detection of bladder injuries [44,45]. Careful and systematic evaluation of all the bladder mucosa should be performed by cystoscopy. Signs of injury, the presence of disruption of mucosal integrity, or suture material should be directly visualized. The suspicion of ureteral injury or obstruction should be raised when ureteral efflux from one or both ureters is not visualized [45]. The decrease in Trendelenburg position, combined with low intra-abdominal pressure, reduces the gravity effect on the ureter, facilitating its peristalsis and function [45]. In some cases, endovenous fluid administration or diuretics could expedite ureteral peristalsis and efflux. However, undiagnosed urological tract malformations, several grades of kidney failure, or more commonly, dehydration, could complicate the procedure, leading to false detection of urinary injuries [45].

Gilmour et al. [46] showed that the sensitivity of cystoscopy for the detection of injury is lower than its specificity. According to these authors, up to 10–15% of injuries are not detected, and sometimes the procedure could provide a false sense of reassurance to the surgeon. In a recent monocentric study [44], universal cystoscopy performed after hysterectomy was compared to selective cystoscopy realized at the surgeon’s discretion. The authors concluded that implementation of universal cystoscopy after hysterectomy leads to a decrease in delayed postoperative urologic complications. On the other hand, a large systematic review and meta-analysis including 79 studies and 41482 hysterectomies showed that universal cystoscopy did not decrease the incidence of delayed genitourinary injuries [47]. Delayed thermal effects, post-ligature swelling, and defects too small to be detected visually could be missed with routine cystoscopy [47]. Furthermore, despite the extremely low risk of the procedure, cystoscopy is linked with urinary tract infections and bladder or urethral trauma. Allergic reaction to intravenous (i.v.) dyes is rare, and a few cases are also described [48]. Moreover, a retrospective study [49] including 101 cystoscopies reported one complication related to the mechanical nature of the procedures. On the contrary, in a large study, authors enrolled two hundred fifty-one women who underwent cystoscopy at the time of hysterectomy, and no complications resulting from this procedure were reported [50]. In addition, the authors commented that the absence of infectious complications associated with routine cystoscopy could be explained by the administration of antibiotic prophylaxis at the time of hysterectomy.

One of the most important arguments against universal cystoscopy is the procedural cost. In a cost analysis published in 2001 [51], 67 cystoscopies would have to be performed to identify a single ureteral injury, and a minimum threshold ureteral injury rate of 2% was found to be cost-effective. The study concluded that cystoscopy would be preserved only when the clinical suspicion of vesical injury was high. Although the rate of late bladder complications is significantly reduced, cystoscopy remains a time- and money-consuming procedure, and its routine use after hysterectomy is not warranted [52]. Rather, in patients with known risk factors for UC or in whom tenacious bladder adherence was found intraoperatively, a bladder catheter for a few days could be considered [53].

### 4.5. Ureteral Identification

Several risk factors are accounted for in ureteral injuries during laparoscopic surgery, such as previous surgeries or cesarian sections [54]. The ureteral course could be distorted by adhesions derived from tumors, endometriosis, voluminous uterine myomas, adnexal masses, and previous urogynecology surgery. The ureter runs along the pelvic cavity by crossing from lateral to medial the bifurcation of the iliac common artery at the pelvic brim, just medial to the ovarian vessels [55]. At the level of the ischial spines, it runs in the broad ligament and flows into the ureteral canal formed by the cardinal ligament. At this point, the ureter is crossed cranially by the uterine artery; this anatomical landmark is about 1.5 cm distant from the cervix [55]. Afterward, it runs medially and enters the bladder close to the anterior vaginal wall. The ureter also lies very next to the uterosacral ligament while running at the base of the broad ligament, and at this level, it can be visualized through the peritoneum [56].

Ureteric injuries occur generally in specific anatomic sites along their course, especially when particular surgical steps are performed during gynecology procedures [45].

The common sites of ureteric wounds are: at the pelvic brim, at the bifurcation of the common iliac artery, at the lateral pelvic wall, at the cross with the uterine artery, and at the level of the ureteric canal [57]. Surgical procedures linked with high ureteral injuries are the clamping of the infundibulopelvic ligament or the internal iliac artery. Pararectal and perivesical space development and parametrial dissection could also lead to medial ureteral wounds. Finally, low ureteral injuries can occur during vaginal fornix clamping during a hysterectomy [58].

The ureteric intramural portion, where the ureter inserts into the bladder trigon, is an uncommon site for injury. At this level, ureteric injuries could occur when the Yabuki space is developed or in the presence of a wide lesion involving the base of the bladder [59]. Most of the injuries (87.1%) were located at the distal third part of the ureter [60] and especially at the cross with the uterine artery [58]. Technical risk factors comprise intraoperative hemorrhage, coexistent bladder injury, and inexperienced surgeons [58]. Intraoperative types of ureteric injuries comprise crushing from misapplication of a clamp, ligation with a suture, partial or complete transection, angulation with secondary obstruction, stripping or electrocoagulation ischemia induction, and resection of a part of the ureter [45]. Post-operative injuries include avascular damage and necrosis, kinking-peritonization of the vaginal stump after hysterectomy, and subsequent obstruction over hematoma or lymphocele [58].

Intraoperative identification of the ureters is a crucial step during many procedures in urology, gynecology, and general surgery. Surgeons should constantly know where the ureter is at all times, and an appropriate approach with adequate exposure is essential, avoiding blind clamping and ligature of blood vessels and limiting the zone of coagulation to reduce thermal injury. Laparoscopic identification of ureters can be difficult due to a loss of tactile sensation and less tissue discrimination resulting from magnified video imaging [61]. Several techniques have been developed to facilitate ureteral identification during pelvic surgery. Some authors proposed the use of indocyanine green (ICG) for intraoperative localization of ureters [62,63]. ICG is a Food and Drug Administration-approved fluorescent anionic amphiphilic tricarbocyanine dye that fluoresces at excitation with near-infrared (NIR) wavelengths (about 750 to 800 nm) [64]. It is applied in several medical fields, from ophthalmic retinal angiography to cardiac and hepatic function testing, but also to determine tissue viability [65].

For accurate and easier localization of ureters during laparoscopic surgery, a preoperatively cystoscopy-guided instillation of ICG dye could be performed; with this technique, ureters are visualized during laparoscopic surgeries using an optical imaging system with invisible NIR light. Mandovra et al. [63] analyzed this technique in different pelvic surgeries. In this study, ICG was administered immediately preoperatively with a 6-Fr ureteral catheter by a cystoscopic guide, and the fluorescence of the ureters was visualized in the NIR mode. Ureteral fluorescence was visualized in all thirty cases analyzed, and it was appreciated until the end of the surgery. No intraoperative or postoperative complications or adverse effects were observed by the authors. In gynecology oncology, this technique was tested by Cabanes et al. [66], who injected 8 mL of ICG solution (concentration 1.25 mg/mL) in the ureter through a 6 Fr ureteral catheter. After the removal of the ureter catheter, intraoperative identification of the ureter course at the level of the infundibulopelvic ligament, transperitoneal, and lateral and ventral parametria was achieved. The authors suggest the use of this approach also during open abdominal surgery, with the use of a specific near-infrared camera. The application of ICG for ureter identification was also implemented in robotic surgery. Siddighi et al. [67] provided a study including patients who underwent robotic sacrocolpopexy. After the dissolution of twenty-five milligrams of ICG in 10 mL of sterile water, the fluorescent dye was injected through a 6-Fr ureteral catheter by cystoscopy. The bilateral ureteral visualization was achieved in all patients, and no complications were reported. Authors assessed that with this technique, gynecologists may be more comfortable compared with ureteral catheterization, and a real-time delineation of the ureter is easily achieved. A viable alternative to ICG ureteral injection is the fiber optic light strands contained within two 5-F transparent ureteral catheters that were at first proposed in urologic surgery [68]. The application of these catheters requires a cystoscopy before the induction of peritoneal insufflation. This technique was later implemented in other surgeries. Miyajima et al. [69] showed that the ureteral illumination tube facilitates a sufficient understanding of the laparoscopic anatomy and the dissection of the proper layer of mesenterium in colorectal surgery. More recent publications [70] indicate that the iatrogenic ureteral injury due to surgery and the conversion rate to laparotomies were minimized with the use of prophylactic illuminated ureteric catheters. Recently, some authors proposed the use of a novel type of fluorescent ureteral catheter during gynecology laparoscopic procedures [71]. A near-infrared ray catheter (NIRC) is a newly developed device that consists of a catheter containing a fluorescent resin that exhibits a fluorescent reaction by irradiation with near-infrared light [72]. The preoperative placement of a bilateral fluorescent ureteral catheter allows the real-time detection of the ureteral course during surgery. In a recent study by Hiroaki et al. [73], the authors used this device in three patients who underwent laparoscopic hysterectomy for uterine myomas. The time of catheter insertion, according to the authors, ranged from 4 to 11 min. Identification and ligation of the uterine arteries, dissection of the cardinal ligament, incision of the vaginal canal, and suturing of the vaginal stump were performed under ureter visualization by near-infrared light, facilitating the real-time confirmation of the ureter positions. After a follow-up of 6 months, no urinary tract infection or injury was found. Following these results, Iori et al. [72] demonstrated the feasibility, safety, and potential usefulness of real-time intraoperative visualization of the ureters using a novel NIRC fluorescent ureteral catheter in laparoscopic hysterectomy for endometrial cancer. These authors suggest that this novel technique could be useful in the cases of obesity, severe pelvic adhesion, deep infiltrating endometriosis, and malignancy in gynecologic laparoscopic surgery. Despite the implementation of these new technologies, their routine employment is limited to selected trials and clinical studies. Most gynecology surgeons approach the pelvis using classic anatomical landmarks and perform a step-by-step surgery to identify all the structures of interest. The key point is still the experience of the surgeon. Education, continued training, and ongoing mentorship represent, nowadays, the only verified strategies to decrease the risk of intraoperative injury [32]. Finally, there is evidence showing that it is needed to complete about twenty laparoscopic hysterectomies with normal anatomy and thirty with large uteri to decrease the risk of ureteral injury at baseline [45].

### 4.6. Pelvic Landmarks and Uterine Manipulation

Many recommendations are relevant to helping reduce the risk of surgical complications during a complex hysterectomy, regardless of the surgical route. Restoration of abnormal anatomy before coagulation and transection of the uterine pedicles is one of the most important. A perpendicular approach to the uterine vascular pedicle during its transaction is another fundamental step [35]. The paravesical space should be developed in cases of difficult and distorted anatomy [59]. This is a retroperitoneal space that lies lateral to the urinary bladder and anterior and superior to the pararectal space and is divided into medial and lateral paravesical spaces by the obliterated hypogastric artery [74]. Dissection of these spaces ensures easy visualization of the uterine artery at its origin from the hypogastric artery and then the ureteral course that runs below the uterine artery [74]. While the lateral paravesical space contains the obturator nerve and lymph nodes, the medial space provides access for bladder dissection; this approach is basic to achieving a complete visualization of the anatomic structures, primarily in conditions of the adherent ureterovesical fold, for example, due to previous cesarean delivery [74].

The retroperitoneal space lying lateral to the rectum on either side is known as the pararectal space. The ureter divides the pararectal space into the medial (Okabayashi’s space) and lateral (Latzko’s space) pararectal spaces [75]. Usually, the lateral pararectal space is developed between the mesoureter and pelvic wall by opening up the space between the internal iliac artery, which lies lateral, and the ureter, which remains medial [76]. The medial pararectal space is otherwise developed between the mesoureter and the rectouterine ligament, where a space between the posterior leaf of the broad ligament (medial) and the ureter (lateral) opens up [76]. The development of these spaces allows the identification of the inferior hypogastric nerve plexus and permits preservation of the visceral afferent and efferent fibers that are directed to the uterus, the vagina, and the bladder [77].

The manipulation of the uterus during laparoscopy hysterectomy is fundamental, as the mobilization of the organ allows surgical access to different anatomical structures. Several types of uterine manipulators were developed to improve the performance of laparoscopic hysterectomy, and numerous models were created to suit the different uterine cavities and shapes of the cervix [78]. An easy mobilization of the uterus for adequate exposure of the operative field: it displaces the uterine arteries cephalad and away from the ureteral course and helps to delineate the proper location of the anterior colpotomy, reducing the risk of bladder injury and minimizing the damage to noble structures in the pelvis [79].

Difficulty in the assemblage, restricted range of uterine motion, the need for cervical dilatation, hard mobilization of big uteri, or a deficiency to maintain a correct pneumoperitoneum are a few disadvantages of uterine manipulators [79]. Uterine rupture is the most frequent complication, but also vaginal wall lacerations, excess hemorrhage, or cervical cup melting are reported [79]. The disintegration of the uterus inside the patient is another complication that raises concern in gynecology oncology surgery, especially in endometrial cancer.

Many studies have analyzed the safety of uterine manipulators in endometrial cancer surgery. Uccella et al. [80], in a retrospective multi-institutional cohort study, evaluated the risk and site of disease recurrence, overall survival, and disease-specific survival in women who had laparoscopic surgery for endometrial cancer, with and without the use of a uterine manipulator. Of the nine hundred and fifty-one patients included, the manipulator was used in 579 patients, while 372 patients were allocated to the no manipulator group. After a follow-up of 46 months, the rate of recurrence was 13.5% in the manipulator group and 11.6% in the no-manipulator groups (*p* = 0.37). The propensity-matched analysis confirmed these findings, and the sites of recurrence were comparable between groups. The type of manipulator and the presence or abscence of a balloon on the tip of the instrument were also included in the analysis, which showed no significant association with the risk of recurrence.

In contrast with these data, in 2020, a Spanish group published an interesting work about the impact of uterine manipulation on oncological outcomes in patients affected by apparent early-stage endometrial cancer [81]. In this retrospective, multicentric study, patients who underwent minimally invasive surgery were included, and the authors evaluated the type of manipulator used, surgical staging, histology, lymphovascular space invasion, FIGO stage, adjuvant treatment, recurrence, and pattern of recurrence. About two thousand and seven hundred patients were included in the study, and in 1756 patients, a uterine manipulator was used. The authors showed that the use of a uterine manipulator in uterus-confined endometrial cancer (stages FIGO I and II) was associated with lower disease-free survival (hazard ratio, 1.74; 95% confidence interval, 0.57–0.97; *p* = 0.027) and a higher risk of death (hazard ratio, 1.74; 95% confidence interval, 1.07–2.83; *p* = 0.026). No differences were found in the pattern of recurrence between the two groups.

Recently, a multicentric randomized Italian trial enrolled patients with early-stage endometrial cancer (G1 and G2) that were randomly divided into two groups according to the use or not of the uterine manipulator [82]. The authors aimed to evaluate the impact of the uterine manipulator on lymph-vascular space invasion (LVSI). Clermont Ferrand’s uterine manipulator was used in one group, while in the no-manipulator group, the cervix was closed with a cross stitch before starting the abdominal surgery. Of the 154 patients enrolled, 76 were in the no-manipulator group, and 78 were in the other one. The analysis showed no statistically significant differences in LVSI between the two study groups. No statistically significant differences were also found in the pattern of LVSI or peritoneal cytology. Likewise, the number of recurrences was similar in the two groups without reaching a statistically significant difference.

Although the risk of uterine manipulator use in gynecology oncology surgery remains unclear, the benefits of its application could be important in particular cases where anatomy is distorted by endometriosis and previous pelvic inflammations from radiation therapy [83]. Furthermore, young surgeons with little experience would benefit from the use of uterine manipulators. Accurate selection of the patients and continued surgical training should permit the right use of uterine manipulators.

## 5. Strength and Limitation

This study provides an updated urologic complication rate in a specific subset of patients with oncologic disease. Unlike other authors, all benign gynecologic diseases were excluded from the analysis. Furthermore, only surgeons dedicated to gynecologic oncology were considered with a recent case history. In this way, the study reports a complication rate updated to the latest guidelines in a recent surgical scenario with present-day devices. On the other hand, the study has limitations inherent in its retrospective nature. A low complication rate is reported; therefore, a net division between intra- and postoperative urinary complications was not statistically possible.

## 6. Conclusions

The urological complication rates in patients with oncological disease are events that occur relatively infrequently in the expert hands of dedicated surgeons. Surgical radicality, advanced FIGO stage, and cases requiring adjuvant treatment are independent factors associated with urinary complications. An update on urologic complications in gynecologic oncologic surgery is needed for better patient counseling and further improvement of surgical techniques.

## Figures and Tables

**Table 1:** Patients’ Characteristics.

	Total	Complication *n*; %	No Complication *n*; %
	260; 100	9; 3.5	251; 96.5
Parity	214	9; 100	195; 77.7
Age median (years)	63 (IQR: 52–73)	50 (IQR: 50–60)	63 (IQR: 53–73)
BMI median (Kg/m^2^)	27 (IQR: 24–30)	28 (IQR: 25–29)	27 (IQR: 24–30)
Hypertension	94	4; 44.4	90; 35.9
Diabetes	20	0; -	20; 8.0
Hypothyroidism	32	1; 11.1	31; 12.4
Cardiovascular disease	27	0; -	27; 10.8
Psychiatric disorders	5	0; -	5; 2.0
ASA Status			
1	12	1; 11.1	11; 4.4
2	204	7; 77.8	197; 78.5
3	44	1; 11.1	43; 17.1
Previous cesarean section	30	2; 22.2	28; 11.2
Previous urologic surgeries	5	1; 11.1	4; 1.6

ASA: American Society of Anesthesiologists. BMI: body mass index. IQR: Interquartile range.

**Table 2:** Pathological data and surgical procedures performed.

	Total *n*; %	Complication *n*; %	No Complication *n*; %
	260; 100	9; 3.5	251; 96.5
Primary disease			
Endometrial cancer	180; 69.2	6; 66.7	174; 69.3
Cervical cancer	18; 6.9	2; 22.2	16; 6.4
Ovarian cancer	9; 3.5	1; 11.1	8; 3.2
BOT	51; 19.6	0; -	51; 20.3
Uterine Sarcoma	2; 0.8	0; -	2; 0.01
LVSI	37; 14.2	3; 33.3	34; 13.6
FIGO Stage			
I	214; 82.3	5; 55.6	209; 83.3
II	46; 17.7	4; 44.4	42; 16.7
Grading			
G1	143; 55.0	2; 22.2	141; 56.2
G2	63; 24.2	4; 44.4	59; 23.5
G3	54; 20.8	3; 33.3	51; 20.3
Simple Hysterectomy	82; 31.5	0;-	82; 32.7
SLN	115; 44.2	4; 44.4	111; 44.2
Pelvic LND	58; 22.3	4; 44.4	54; 21.5
Aortic LND	38; 14.6	3; 33.3	35; 13.9
C1 Hysterectomy	5; 1.9	2; 22.2	3; 1.2
Adjuvant treatment	87; 33.5	6; 66.7	81; 32.3

BOT: borderline ovarian tumor. FIGO: International Federation of Gynaecology and Obstetrics. LND: Lymphadenectomy. LVSI: lymphovascular space invasion. SLN: sentinel lymph node.

**Table 3:** Complication type and management.

Intraoperative		Type	Treatment	Clavien Dindo
	1	Ureteral resection	Uretero-ureteral anastomosis and ureteral catheterization	-
	3	Superficial vescical injury	Suture and bladder catheterization for 3 days	-
	1	Complete vescical perforation	Suture and bladder catheterization for 7 days	-
Postoperative	1	Ureteral stricture with hydronephrosis	Ureteral catheterization	IIIA
	1	Ureteral fistula	Ureteral catheterization	IIIA
	1	Vesicovaginal fistula	Martius labial interposition flap	IIIB
	1	Bladder stupor	Bladder catheterization for 7 days	II

**Table 4:** Univariable logistic regression.

Characteristics	OR	95% CI	*p*-Value
*Clinical data*			
Age	0.97	0.93–1.02	0.2
BMI	1.06	0.95–1.16	0.2
Parity	NA	0.00–NA	>0.9
Hypertension	1.43	0.35–5.54	0.6
Hypothyroidism	0.89	0.05–5.08	>0.9
ASA Status	0.54	0.14–2.30	0.4
Previous cesarean section	2.28	0.33–9.98	0.3
Previous urologic surgery	1.98	0.72–3.92	0.082
*Primary disease*			
Endometrial cancaer	0.89	0.23–4.28	0.9
Cervical cancer	4.20	0.59–19.1	0.089
Ovarian cancer	0.41	0.02–2.28	0.4
Sarcoma	0.00	NA	>0.9
Borderline Ovarian Tumor	0.00	NA	>0.9
*Pathological data*			
LVSI	3.19	0.65–12.7	0.11
FIGO Stage	3.98	0.95–15.7	0.046
Tumor grading	1.95	0.89–4.45	0.10
*Surgery Performed*			
C1 Hysterectomy	23.6	2.79–167	0.001
Sentinel lymph node biopsy	1.01	0.24–3.90	>0.9
Pelvic lymphadenectomy	2.92	0.70 -11.4	0.12
Aortic lymphadenectomy	3.09	0.63–12.3	0.12
Adjuvant treatment	4.20	1.08–20.3	0.046

ASA: American Society of Anesthesiologists. BMI: body mass index. CI: confidence interval. FIGO: Federation International Gynecologic and Obstetrics. LVSI: lymphovascular space invasion. NA: not applicable. OR: odds ratio.

## Data Availability

Data will be provided by the corresponging author upon reasonable request.
